# Setting priorities for the health care sector in Zimbabwe using cost-effectiveness analysis and estimates of the burden of disease

**DOI:** 10.1186/1478-7547-6-14

**Published:** 2008-07-28

**Authors:** Kristian Schultz Hansen, Glyn Chapman

**Affiliations:** 1 Institute of Public Health, Department of Health Services Research, University of Aarhus, Vennelyst Boulevard 6, DK-8000, Aarhus C, Denmark; 2 DBL-Institute for Health Research and Development, Jaegersborg Alle 1D, DK-2920, Charlottenlund, Denmark; 3 IMMPACT, University of Aberdeen, 2nd Floor, Foresterhill Lea House, Westburn Road, Aberdeen, AB25 2ZY, UK

## Abstract

**Background:**

This study aimed at providing information for priority setting in the health care sector of Zimbabwe as well as assessing the efficiency of resource use. A general approach proposed by the World Bank involving the estimation of the burden of disease measured in Disability-Adjusted Life Years (DALYs) and calculation of cost-effectiveness ratios for a large number of health interventions was followed.

**Methods:**

Costs per DALY for a total of 65 health interventions were estimated. Costing data were collected through visits to health centres, hospitals and vertical programmes where a combination of step-down and micro-costing was applied. Effectiveness of health interventions was estimated based on published information on the efficacy adjusted for factors such as coverage and compliance.

**Results:**

Very cost-effective interventions were available for the major health problems. Using estimates of the burden of disease, the present paper developed packages of health interventions using the estimated cost-effectiveness ratios. These packages could avert a quarter of the burden of disease at total costs corresponding to one tenth of the public health budget in the financial year 1997/98. In general, the analyses suggested that there was substantial potential for improving the efficiency of resource use in the public health care sector.

**Discussion:**

The proposed World Bank approach applied to Zimbabwe was extremely data demanding and required extensive data collection in the field and substantial human resources. The most important limitation of the study was the scarcity of evidence on effectiveness of health interventions so that a range of important health interventions could not be included in the cost-effectiveness analysis. This and other limitations could in principle be overcome if more research resources were available.

**Conclusion:**

The present study showed that it was feasible to conduct cost-effectiveness analyses for a large number of health interventions in a developing country like Zimbabwe using a consistent methodology.

## Background

There is an increasing number of cost-effectiveness studies aiming at analysing the *allocative *efficiency of the health care sector. These analyses incorporate costs and effects of interventions directed at different health problems and different patient groups and often include a large number of interventions. Examples from developed countries include analyses performed in United Kingdom [1], Australia [2] and in Oregon State in the USA [3] while a large database on cost-effectiveness analyses from all over the world is maintained by an American university [4]. For developing countries, the World Bank health sector priorities review [5-7] assessed the costs and effectiveness of health interventions directed at major health problems for low and middle income regions of the world. In a similar effort, the World Health Organization estimated costs per DALY for a wide range of health interventions for 14 epidemiologic sub regions and in addition developed tools enabling individual countries to perform similar cost-effectiveness analyses based on local estimates on e.g. disease burden and unit costs of various health services [8-10]. At an individual country level, a list of costs per discounted life year gained for a large number of preventive and curative health interventions was developed for Guinea [11].

While such cost-effectiveness analyses aiming at assessing allocative efficiency may be very useful for setting priorities in the health care sector of a given country, several features of this technique have been identified as being problematic. Since these analyses often include a large number of health interventions, these exercises are extremely data intensive in terms of estimating the required information on costs and effectiveness. Consequently, simplifying assumptions and shortcut methods have been applied in order to make the data collection task more manageable. For instance, it is often assumed that health interventions are produced under constant returns to scale so that the costs per health output do not vary with the scale at which the intervention is undertaken thus making it necessary only to estimate a single point on the cost function [6,12]. It is also common practice to exclude important cost categories such as costs borne by patients [13]. Further, required information may be predicted using statistical models rather than actual data collection [9]. A major concern is the severe lack of information on effectiveness of health interventions [14]. Finally, concerns have been raised over the relevance and applicability to priority setting in a particular country of the published allocative cost-effectiveness analyses since these have often been developed as regional estimates [15].

Presently, there is not much knowledge of the relative cost-effectiveness of health services offered in the Zimbabwean public health care sector. Such information may however be useful for assessing the efficiency of resources used in a situation of dwindling health care funds and steeply increasing demand. The main objective of this paper is therefore to provide input into an analysis of identifying ways of improving the allocative efficiency of resource utilisation in the health care sector of Zimbabwe. The general research strategy for achieving this objective is inspired by the approach previously utilised by the World Bank [5,6,16,17]. As a first step, this approach entails the estimation of the level of ill-health of the Zimbabwean population in 1997 using DALYs as the societal health outcome measure. Results of this component have been reported elsewhere [18] and key figures describing the burden of disease by cause in 1997 have been reproduced in Annex 1 of the present paper. The second step involves the estimation of costs per DALY gained for a large number of health interventions followed by the development of essential packages of health interventions which address large amounts of ill-health at low costs. The present paper focuses on the second step. In addition, having finalised this kind of analysis in Zimbabwe, this study also provides an opportunity to discuss the feasibility of conducting this very data intensive World Bank approach in a developing country setting.

### The context of the health care system

At the time of this study, the disease pattern in Zimbabwe is heavily dominated by communicable, maternal, perinatal and nutritional conditions [19] similar to other countries in Sub-Saharan Africa although Zimbabwe is plagued with an unusually large disease burden due to HIV (Annex 1). The health of the nation has traditionally been a high priority and large investments in the public health care sector in the 1980s led to impressive improvements in key health indicators although the years following 1990 saw a reversal in most health indicators [20] – a development further exacerbated in more recent time due to decreasing GDP, dwindling health care funds and massive emigration of health sector personnel [21]. The health care sector is a highly heterogeneous section of the economy. Provision of health care services is offered by government, church missions and other NGOs, industries and mines, private practitioners and traditional healers. Measured by the number of health facilities, government is the single most important provider [22]. Private practitioners and hospitals are relatively abundant in larger cities where these providers are able to attract large proportions of the available health personnel. Government of Zimbabwe has succeeded in organising its own institutions as well as church mission facilities and some of the private sector facilities into a four-tiered system of health care service delivery. Health centres manned by qualified nurses are the first level followed at the next levels by district, provincial and central hospitals where hospital services of increasing complexity are offered requiring more specialised personnel and equipment. The head office of the Ministry of Health and Child Welfare constitutes the highest level of the public health care sector and it is the main actor in terms of health policy making and development. For instance, the head office is responsible for the allocation of all government health care funds among health facilities as well as steering important processes such as the Zimbabwe Essential Drugs Action Programme (ZEDAP) which results in a list specifying the most cost-effective drugs for a large number of health problems [23] that is used extensively by all health facilities in the country.

## Methods

### Choice of interventions for the cost-effectiveness analyses

Curative interventions for the present study included the treatment of common health problems at hospital inpatient and outpatient departments as well as health centres. These interventions covered both single treatment episodes and more long term management of chronic conditions. Preventive interventions included five vertical activities: residual house spraying to prevent malaria, immunisation of children (measles, polio, tuberculosis, diphtheria, pertussis and tetanus), surveillance and targeted supplementary feeding of wasted children, HIV prevention through improved access to treatment of sexually transmitted infections (STIs) and health promotion of personal and domestic hygiene in order to decrease the incidence of diarrhoeal diseases.

### Cost data collection and unit costs estimation at selected study sites

In order to estimate the costs of individual curative and preventive health interventions, a number of public health providers were visited for the collection of the necessary cost data. Study sites were randomly chosen from all over the country. With respect to curative health interventions, six health centres out of a total of around 1200 were selected for the cost analysis. Health centres offered outpatient services and selected preventive activities such as immunisation. Five district level hospitals including two mission hospitals from a total of 130 hospitals were sampled for the costing of inpatient services, surgical procedures and outpatient services. Finally, two provincial hospitals (from a total of 8) were randomly selected and these offered similar services as district hospitals but the former hospitals were able also to provide more specialised services. The highest level, central hospitals, was excluded from the costing analysis. Preventive interventions were organised in a vertical fashion involving provincial health offices and district hospitals as well as services performed by health facilities (e.g. vaccinations at health centres and hospitals). Two provinces out of a total of eight were randomly chosen and two districts were randomly selected within each province (a total of 14 districts). Finally, the Ministry of Health Headquarters and two provincial health offices were visited to capture additional programme costs of curative and preventive interventions such as central purchasing of drugs and high level administrative personnel [10].

The costing perspective taken for this study was the health provider's view (Ministry of Health and Child Welfare) since the objective of the present cost-effectiveness analysis was to determine how the largest slice of the burden of disease could be cut using a given government budget [24].

Activities at each study site incorporated the identification, measurement and subsequent valuation of resources required to offer health services. Government accounting systems provided at each study site the level of actual, recurrent expenditure by category including for example salaries by type of personnel, stationery, electricity, maintenance and drugs. With respect to capital inputs at each study site, a quantity surveyor estimated the present day construction costs per square metre by type of office or department. Further, a list of available equipment and furniture was developed and subsequently valued using market prices. From these replacement costs of buildings, equipment and furniture, an annual equivalent was calculated using the annuitization method [24,25] assuming a real discount rate of 3% and expected life spans of 30, 7 and 10 years for the mentioned capital inputs.

These costs by category were at each study site allocated to the health interventions selected for this study. This was done by applying the standard step-down costing methodology [24,26] consisting initially of categorising activities (in practice wards and departments) in a study site into a hierarchical system with the final product (such as patient care) at the lowest level and with support and overhead activities at successively higher levels. Subsequently, the aggregate costs by category were allocated to final activities in a step-wise fashion using simultaneous equation techniques [[24], Ch. 4] and the development of allocation criteria reflecting actual resource use. At the end of the standard step-down costing procedure, all costs of a study site had been distributed to the final service departments so that an average costs figure could be calculated by dividing the number of services provided by individual departments. Micro-costing techniques [27] were used to supplement the above information in order to achieve information on interventions against individual diseases. For instance, a review of a sample of inpatient notes was performed at hospitals in order to capture the treatment pattern of the most common health problems. With respect to the treatment of the less common health problems, official treatment guidelines were used [23].

Having finalised the study activities described above, unit costs of individual curative and preventive services were available for the study sites included.

### Costs of interventions at population level

Unit costs of individual health interventions estimated from data collected at the study sites were utilised for calculating the total costs of offering this service for a population group as a whole. This was done to take account of the fact that costs and effects measured in DALYs averted depended on age of onset of disease. The total costs of a specific curative health intervention were calculated for a hypothetical district population of 250,000 individuals in Zimbabwe with the same age and sex distribution and incidence of diseases as the country as a whole. The number of treatments for each disease was determined by incidence and the health seeking behaviour of the population. Information on incidence of diseases was drawn from a national study which provided estimates of new cases of disease by age and sex groups for the year 1997 [18]. In addition, the proportions of cases by disease likely to seek treatment were determined based on advice from clinical experts as well as the National Health Information System [19]. Using these two types of information, the total number of treatments by age and sex could be estimated for each disease under study. Subsequently, the total costs of a curative health intervention were estimated by multiplying this number with the relevant unit costs:

(1) $$C^{j}=U^{j}\sum_{a}\sum_{s}I_{as}^{j}H_{as}^{j}$$

where *C*^*j *^is the population level costs of intervention *j*, *U*^*j *^indicates the unit costs of curative health intervention *j*. In addition, $I_{as}^{j}$ is the absolute, annual number of incident cases of a health problem (which may be treated by intervention *j*) in population group of age *a *and sex *s *while $H_{as}^{j}$ is the proportion of incident cases seeking treatment in the same population group. Outpatient services were offered both at health centres and hospitals. It was assumed that 80% of all cases were treated at health centres and 20% at district hospital outpatient departments corresponding to the actual health seeking behaviour [19]. Some health problems required life long treatment like for instance insulin-dependent diabetes. In these cases, the specific cost figures estimated for a given length of time were recalculated to match the life expectancies at various ages of onset of the disease as indicated in the formula below:

(2) $$C^{j}=\sum_{a}\sum_{s}I_{as}^{j}H_{as}^{j}\sum_{t=1}^{T(as)}[{\left(1+r\right)}^{-(t-1)}A_{t}^{j}]$$

where $A_{t}^{j}$ is the annual costs at time *t *for health intervention *j *for a chronic condition while *T*(*as*) indicates the life expectancy of an individual belonging to population group of age *a *and sex *s*. Future costs were discounted using a real discount rate *r *of 3 percent.

The primary preventive interventions incurred costs at district and provincial health offices and typically also at the level of health providers such as health centres and hospitals. The pattern of cost components for preventive interventions therefore followed the general form:

(3) $$C^{j}=D^{j}+P^{j}+U^{j}\sum_{a}\sum_{s}M_{as}^{j}N_{as}^{j}$$

where *D*^*j *^and *P*^*j *^represent the overall costs related to preventive intervention *j *at the district and the particular district's share of the provincial office respectively. In addition, *U*^*j *^denotes the unit costs of preventive activities such as vaccinations or STI treatments performed at health centres and hospital outpatient departments. Finally, $M_{as}^{j}$ is the absolute number of individuals in population group of age *a *and sex *s *targeted for intervention *j *and with $N_{as}^{j}$ denoting the percentage actually covered. Information on the number of individuals in each age and sex group in the study population could be obtained from the most recent census [28,29] and updating these figures using estimates of population growth [30]. Coverage of the five preventive health interventions was established through discussions with the responsible staff in the four districts. For some activities such as immunisation, information on coverage was collected as part of a recent Demographic and Health Survey [31].

### Estimation of effectiveness of interventions at population level

The benefits of an intervention were measured as the reduction in the burden of disease (DALYs averted) as a result of the intervention. Following the Global Burden of Disease methodology [32-34], the burden of disease for an individual of sex *s *dying prematurely at age *a*, *BOD*_*as*_, and with life expectancy *T(as) *(or suffering from a disease episode starting at age *a *with length *T(as)*) could be calculated from the formula:

(4) $$BOD_{as}=\int_{t=a}^{a+T(as)}WKte^{-\beta t}e^{-r(t-a)}dt$$

where *W *is a quality adjustment factor (disability weight) representing different levels of health [[33,35]: Annex 3]. The component *Kte*^-*βt *^is an age weighting curve of an inverted u-shape so that the relative value of life years in young adulthood is higher than in other ages while *e*^-*r*(*t*-*a*) ^is the discount factor using discount rate *r *= 0.03. Finally, rather than using actual life expectancies of the population under study, the DALY methodology employs long life expectancies from a low mortality model life table (Coale-Demeny West Level 26 [36]). Life expectancies *T(as) *therefore depend on both age and sex. The benefit in terms of DALYs gained from a successful intervention *j *for a person of age *a *and sex *s *is calculated in the following way:

(5) $$\Delta BOD_{as}^{j}=BOD_{as}-BOD_{as}^{j}$$

where $BOD_{as}^{j}$ is the burden of disease after a successful intervention. For instance, the number of DALYs gained for an individual dying prematurely at age *a*_1 _without treatment but postponing death until age *a*_2 _(*a*_1 _<*a*_2_) following an intervention can be calculated using equation (5). A detailed explanation using worked examples of how to calculate DALYs for cost-effectiveness analysis has been presented by Fox-Rushby and Hanson [37].

Effectiveness of health interventions in a real world setting depend on a wide range of factors [11,38]. Four factors were identified for the present study as having an important influence on the effect of curative interventions: efficacy of individual drugs, diagnostic accuracy, appropriateness of the treatment prescribed and patient compliance.

With respect to efficacy, sources of information for this measure by type of drug were mainly a World Bank review [5], Cochrane systematic reviews (such as for instance trachoma [39]) or articles identified through the Cochrane register of randomized controlled trials. Very little hard evidence from the Zimbabwean setting could be found on the other three factors so estimates of these aspects were determined for each health problem based on discussions with clinical experts. In a similar fashion as applied by Evans *et al*. [13], the effectiveness of a health intervention was estimated by reducing the efficacy of the relevant drug by a factor between 0 and 1. The benefits at population level in terms of DALYs averted of a specific curative health intervention *j *were subsequently calculated as:

(6) $$DALYs^{j}=E^{j}B^{j}F^{j}G^{j}\sum_{a}\sum_{s}I_{as}^{j}H_{as}^{j}\Delta BOD_{as}^{j}$$

where *E*^*j*^, *B*^*j*^, *F*^*j *^and *G*^*j *^are efficacy of the drug prescribed, diagnostic accuracy, correct treatment and patient compliance respectively for curative intervention *j *measured as percentages. Expressed in words, this equation estimates the number of individuals cured through treatment *j *by excluding ineffective services from the total number of individuals seeking treatment and translating the resulting health benefits into DALYs averted.

A similar procedure was applied to preventive interventions involving first determining the effect under ideal conditions followed by adjusting this to incorporate real world conditions. Efficacy of malaria spraying was derived from a study in South Africa which compared the prevalence of malaria infection in sprayed areas and non-sprayed areas [40]. Similarly, efficacy estimates were derived for environmental health [41-43], food supplementation [44], vaccines [45,46] and STI syndromic management [47,48]. The number of DALYs averted at population level for a given preventive intervention *j *was calculated as:

(7) $$DALYs^{j}=E^{j}R^{j}\sum_{a}\sum_{s}I_{as}^{j}\Delta BOD_{as}^{j}$$

where *E*^*j *^is the efficacy of the intervention under ideal circumstances and *R*^*j *^is any necessary downward adjustment (less than perfect coverage) of efficacy while $I_{as}^{j}$ is the incidence of disease in different age- and sex groups. Coverage of the five preventive health interventions was established through discussions with the responsible staff in the four districts included or in the case of EPI utilising the Demographic and Health Survey [31].

### Calculation of cost-effectiveness ratios

Having estimated the total costs and effectiveness of various health interventions, the cost-effectiveness ratio for intervention *j*, *CER*^*j*^, was found as:

(8) $$CER^{j}=\frac{C^{j}}{DALYs^{j}}$$

where costs were estimated using equation (1), (2) or (3) and effects were estimated using (6) or (7).

### Development of essential health packages

The selection of health interventions for essential health packages may be done by applying different sets of principles. According to the World Bank principles for developing health packages [16], desirable health interventions are those with low cost-effectiveness ratios and at the same time address important health problems. Another possible set of principles is a pure cost-effectiveness criterion [49]. This entails utilising a process consisting of selecting first the intervention with the lowest cost-effectiveness ratio and then calculating the total costs of averting this health problem. The subsequent step chooses the intervention with the second lowest cost-effectiveness ratio and also calculating the total costs of averting this health problem and so on until the budget is exhausted.

Assuming that the cost-effectiveness ratios estimated for the health interventions of this study complied with the assumptions of perfect divisibility and constant returns to scale [50,51], the total costs and effects in terms of disease reduction of various sets of interventions could be estimated. Median cost-effectiveness ratios were utilised for each type of intervention. Estimates of the burden of disease by cause which must be addressed by the selected interventions were obtained from a previous national study [18] and reproduced in Annex 1. It was further assumed that 300 millions of Zimbabwe dollars were available for the essential packages. This corresponded to just below 10 percent of actual capital and recurrent expenditure at the national level in the financial year 1997/1998. Two additional restrictions were imposed. First, the majority of diseases could not be averted at a single level of the health system. For instance, if an intervention against pneumonia was selected to be part of the package, this health problem could not be fully avoided by offering treatment through health centres. It would be necessary to offer hospital treatment as well. Secondly, it was assumed that at most 30 percent of the HIV burden could potentially be averted through the preventive intervention included in the study (STI treatment) to avoid the budget being exhausted by this single intervention.

### Sensitivity analysis

The sensitivity of the cost-effectiveness ratios was assessed by varying important parameters and assumptions. Instead of a 3% discount rate utilised for the baseline calculations of cost-effectiveness ratios, these were recalculated using discount rates of 6 and 10%. Estimated time preferences with respect to life years vary a lot [52] although a recent empirical study in Tanzania suggested a time preference of a similar size to the range chosen above [53]. The size of the discount rate affected both the effects of health interventions through the DALY formula and the costs of health services. The long life expectancies from the chosen model life table were replaced by actual, period life expectancies as recommended in the recent World Bank health sector priorities review [12]. Much shorter, actual Zimbabwean life expectancies were estimated based on the population size and the number of deaths by age and sex obtained from the census of 1997 [30]. Furthermore, rather than the inverted u-shape of the age weighting function suggested by the DALY methodology, an age weighting function with an equal value of 1 on each life year was used as also suggested by the World Bank health sector priorities review [12]. Some of the health facilities visited operated considerably below full capacity during the study year thus pushing up costs of services. For the sensitivity analysis, cost-effectiveness ratios were recalculated under the assumption that all health facilities were moderately and significantly better utilised (e.g. 80 and 95% bed occupancy rates in inpatient departments respectively). Finally, assessing the robustness of the cost-effectiveness ratios to changes in the effectiveness of interventions was very important since this was an area with little hard evidence. Therefore, cost-effectiveness ratios were recalculated assuming a lower effectiveness of individual health interventions than in the baseline calculations. Calculations were performed utilising effectiveness estimates that were 90, 70 and 50% of the baseline estimates.

## Results

Combining the estimated costs and effects resulted in a list of costs per DALY averted for a large number of health interventions. Table 1 displays the range of costs per DALY of the health interventions included in this study. The specification of a range of costs for individual health interventions reflects the fact that the costs of individual treatments differed in the health facilities and the various preventive programmes due to such factors as varying treatment patterns for similar diseases, availability of resources, degree of capacity utilisation of health facilities and incidence of diseases.

**Table 1:** Costs per DALY in Z$ by type of intervention (Z$17 = US$1), Zimbabwe, financial year 1997/1998

		----- Costs per DALY -----		
	
Disease group and intervention	Level of delivery	Median	Lowest	Highest
HIV				
Prevention through access to treatment for STIs	Vertical programme	129	124	133
Prophylaxis to prevent opportunistic diseases	District hospital, health centres	9,959	9,712	11,063
Prophylaxis to prevent opportunistic diseases	Provincial hospital, health centres	12,473	11,257	13,777

MALARIA				
Treatment as an outpatient	Health centres, hospitals	159	127	214
Treatment as an inpatient	District hospital	173	141	257
Prevention through residual house spraying	Vertical programme	185	81	466
Treatment as an inpatient	Provincial hospital	287	225	327

PNEUMONIA				
Treatment as an outpatient	Health centres, hospitals	42	35	53
Treatment as an inpatient	District hospital	130	102	182
Treatment as an inpatient	Provincial hospital	295	258	317

DIARRHOEAL DISEASES				
Treatment an outpatient	Health centres, hospitals	70	52	102
Prevention through hygiene promotion	Vertical programme	1,011	985	1,054

DYSENTERY				
Treatment as an outpatient	Health centres, hospitals	85	77	102
Treatment as an inpatient	District hospital	531	461	757
Treatment as an inpatient	Provincial hospital	908	814	1,002

TUBERCULOSIS				
Six months treatment with DOTS	District hospital, health centres	815	706	878
Six months treatment with DOTS	Provincial hospital, health centres	973	907	1,085

MALNUTRITION				
Surveillance and targeted food supplementation	Vertical programme	1,489	1,457	1,524

MENINGITIS				
Treatment as an inpatient (bacterial men.)	District hospital	281	266	408
Treatment as an inpatient (bacterial men.)	Provincial hospital	488	404	580
Treatment as an inpatient (meningococcal men.)	District hospital	98	89	144
Treatment as an inpatient (meningococcal men.)	Provincial hospital	176	147	208

PELVIC INFLAMMATORY DISEASE				
Treatment as an outpatient	Health centres, hospitals	365	346	411
Treatment as an inpatient	District hospital	3,666	3,083	4,286
Treatment as an inpatient	Provincial hospital	5,362	3,181	7,543

SYPHILIS				
Treatment as an inpatient	District hospital	3,725	3,429	4,514
Treatment as an inpatient	Provincial hospital	7,612	6,263	9,126

URETHRAL DISCHARGE IN MALE				
Treatment as an outpatient	Health centres, hospitals	13,390	12,599	15,277

VAGINAL DISCHARGE				
Treatment as an outpatient	Health centres, hospitals	3,495	3,312	3,955

CHILDHOOD CLUSTER DISEASES				
Prevention through vaccination	Vertical programme	586	506	700

SCHISTOSOMIASIS HAEMATOBIUM				
Treatment as an outpatient	Health centres, hospitals	259	160	317

SCHISTOSOMIASIS MANSONI				
Treatment as an outpatient	Health centres, hospitals	431	267	528

TRACHOMA				
Treatment as an outpatient	Health centres, hospitals	154	150	165

BACTERIAL CONJUNCTIVITIS				
Treatment as an outpatient	Health centres, hospitals	235	219	271

GLAUCOMA				
Treatment as an inpatient	District hospital	211	160	342
Treatment as an inpatient	Provincial hospital	419	340	508

MINOR COMPLICATIONS IN DELIVERY				
Surgery	District hospital	515	408	717
Surgery	Provincial hospital	757	443	1,072

MAJOR COMPLICATIONS IN DELIVERY				
Surgery (caesarean section)	District hospital	771	591	1,214
Surgery (caesarean section)	Provincial hospital	935	621	1,249

APPENDICITIS				
Surgery	District hospital	606	531	1,136
Surgery	Provincial hospital	1,007	757	1,138

INGUINAL HERNIA				
Surgery	District hospital	314	201	610
Surgery	Provincial hospital	562	557	567

ABSCESS ON EXTREMITY				
Surgery	District hospital	954	837	1,796
Surgery	Provincial hospital	1,555	1,175	1,761

DIABETES MELLITUS				
Inpatient stay and follow-up at health centres	District hospital, health centres	4,999	4,881	5,330
Inpatient stay and follow-up at health centres	Provincial hospital, health centres	5,157	4,749	5,565

HYPERTENSION				
Inpatient stay and follow-up at health centres	District hospital, health centres	8,510	8,419	9,032
Inpatient stay and follow-up at health centres	Provincial hospital, health centres	9,732	9,094	10,370

RHEUMATIC FEVER				
Inpatient stay and follow-up at health centres	District hospital, health centres	3,241	3,012	3,387
Inpatient stay and follow-up at health centres	Provincial hospital, health centres	3,935	3,777	4,093

GASTRITIS				
Treatment as an outpatient	Health centres, hospitals	394	362	461
Treatment as an inpatient	District hospital	1,292	1,203	2,007
Treatment as an inpatient	Provincial hospital	2,465	1,999	2,989

PEPTIC ULCER				
Treatment as an outpatient	Health centres, hospitals	95	75	130
Treatment as an inpatient	District hospital	428	397	684
Treatment as an inpatient	Provincial hospital	850	686	1,036

GASTROENTERITIS				
Treatment as an outpatient	Health centres, hospitals	331	279	425
Treatment as an inpatient	District hospital	895	830	1,287
Treatment as an inpatient	Provincial hospital	1,545	1,295	1,825

SCABIES				
Treatment as an outpatient	Health centres, hospitals	1,074	890	1,403

IMPETIGO				
Treatment as an outpatient	Health centres, hospitals	15,745	13,256	20,249

BODY RINGWORM				
Treatment as an outpatient	Health centres, hospitals	17,266	13,920	23,130

TONSILLITIS				
Treatment as an outpatient	Health centres, hospitals	53,087	49,429	61,289

Among the interventions with the lowest cost-effectiveness ratios, curative treatment at health centres and hospital outpatient departments of pneumonia, severe diarrhoeal diseases, peptic ulcer, dysentery, malaria, trachoma, schistosomiasis haematobium and glaucoma were identified. In addition, curative treatments for meningococcal meningitis, pneumonia and malaria at district and provincial hospitals were also in the group of interventions with low costs per DALY. Preventive interventions of low costs per DALY included improved STI treatment to avert new HIV infections and residual house spraying to avoid malaria infection. In the middle range of the list of costs per DALY averted, fewer interventions based at the first level of the delivery system were found. Treatment of scabies at health centres and hospital outpatient departments was the only exception. With respect to district and provincial hospitals, the treatment of dysentery, peptic ulcer, six months tuberculosis course, complicated deliveries with minor or major surgery (caesarean section) and appendectomy were estimated to have costs per DALY averted in the middle range. Hygiene promotion in the community to prevent diarrhoea was also in the middle range at costs per DALY averted of Z$1,011. The least cost-effective interventions of the list incorporated only health facility-based curative interventions. Health centre and hospital outpatient department interventions incorporated treatment for urethral discharge in males, body ringworm, impetigo and tonsillitis. The reasons for the high costs of these interventions were that these conditions were mild, often self-resolving and the treatment was in some cases not very effective due to low efficacy of the drug recommended. At hospitals, treatment interventions aimed at insulin-dependent diabetes, prophylaxis to avert opportunistic infections in HIV/AIDS patients, hypertension, pelvic inflammatory disease and syphilis were estimated to have high costs per DALY.

The estimated cost-effectiveness ratios were utilised to develop a package of health interventions by applying the World Bank criteria for selection of essential activities (cost-effective and addressing important health problems). As displayed in Table 2, these criteria suggested a variety of health interventions aimed at relieving and averting the health problems due to HIV, pneumonia, diarrhoeal diseases, protein-energy-malnutrition, meningitis, malaria, complicated deliveries and tuberculosis. Potentially, these interventions could avert 26.4 percent of the burden of disease in 1997 at total costs of Z$300 million. The above calculations indicated that relatively few additional resources could address a large percentage of the present burden of disease.

**Table 2:** Burden of disease averted and total costs of a package of health interventions selected based on cost-effectiveness and relative importance of disease criteria, Zimbabwe, financial year 1997/1998

Disease group	Intervention	Level of delivery	% of 1997 disease burden averted	Total costs in millions of Z$
HIV	Prevention	Vertical programme	14.6	93.1
Pneumonia	Treatment	Outpatient care, inpatient care	2.7	19.1
Diarrhoeal diseases	Treatment	Outpatient care	2.4	8.5
Malaria	Treatment/ prevention	Outpatient care, inpatient care, vertical programme	1.8	16.0
Tuberculosis	Treatment	Inpatient care, DOTS	1.6	51.9
Complicated delivery	Treatment	Inpatient care	1.0	35.1
Meningitis	Treatment	Inpatient care	0.9	12.3
Dysentery	Treatment	Outpatient care, inpatient care	0.7	17.8
Protein-energy malnutrition	Prevention	Vertical programme	0.6	46.2
				
Total			26.4	300.0

Using instead a selection procedure based on a pure cost-effectiveness criterion resulted in the interventions displayed in Table 3. Inclusion of health interventions for this package was continued until the total costs were at an identical expenditure level as the package described above. Interventions addressing important health problems like HIV, pneumonia, diarrhoeal diseases, malaria, tuberculosis and complicated deliveries were included in this package as was the case of the package described above. However, instead of including supplementary feeding against malnutrition, this package included relatively minor health problems like bacterial conjunctivitis, gastritis, childhood cluster diseases, glaucoma, peptic ulcer, schistosomiasis and trachoma. The total package could potentially avert a slightly higher share of the 1997 disease burden than the above package (27.2 versus 26.4 percent).

**Table 3:** Burden of disease averted and total costs of a package of health interventions selected based on a pure cost-effectiveness criterion, Zimbabwe, financial year 1997/1998

Disease group	Intervention	Level of delivery	% of 1997 disease burden averted	Total costs in millions of Z$
HIV	Prevention	Vertical programme	14.6	93.1
Pneumonia	Treatment	Outpatient care, inpatient care	2.7	19.1
Diarrhoeal diseases	Treatment	Outpatient care	2.4	8.5
Malaria	Treatment/ prevention	Outpatient care, inpatient care, vertical programme	1.8	16.0
Tuberculosis	Treatment	Inpatient care, DOTS	1.4	45.3
Meningitis	Treatment	Inpatient care	0.9	12.3
Dysentery	Treatment	Outpatient care, inpatient care	0.7	17.8
Complicated delivery	Treatment	Inpatient care	0.5	15.5
Gastritis	Treatment	Outpatient care, inpatient care	0.4	24.9
Trachoma	Treatment	Outpatient care	0.4	2.7
Glaucoma	Treatment	Inpatient care	0.4	4.8
Gastroenteritis	Treatment	Outpatient care, inpatient care	0.3	15.7
Inguinal hernia	Treatment	Inpatient care	0.1	2.7
Childhood cluster	Prevention	Vertical programme	0.1	3.9
Peptic ulcer	Treatment	Outpatient care, inpatient care	0.1	2.8
Pelvic inflam. disease	Treatment	Outpatient care, inpatient care	0.1	9.6
Appendicitis	Treatment	Inpatient care	0.1	3.4
Bacterial conjunctivitis	Treatment	Outpatient care	0.1	1.0
Schistosomiasis	Treatment	Outpatient care	0.1	1.0
				
Total			27.2	300.0

Generally, the cost-effectiveness ratios presented in the tables suggested that there was a good potential for improving the allocative efficiency in the public health care sector of Zimbabwe. Four general strategies could be outlined. Firstly, there were great differences in the estimated cost-effectiveness ratios placed at the top and at the bottom end of the cost per DALY league table for Zimbabwe (Table 1). In principle, therefore, substantial improvements in the allocative efficiency could be achieved by a reallocation of resources from the interventions with high cost-effectiveness ratios to interventions with low cost-effectiveness ratios. Secondly, it appeared beneficial to put particular attention to a narrow range of interventions with low cost-effectiveness ratios. The calculations behind the development of the two intervention packages (Tables 2 and 3) indicated that as much as one quarter of the total burden of disease could be averted by focusing on a few interventions at a level of total costs corresponding to ten percent of the current, national expenditure at that time. Thirdly, the cost-effectiveness figures estimated also confirmed the findings of other studies (e.g. [54,55]) namely that for the same disease, it was more attractive from an efficiency point of view to have the health problem taken care of at the lowest level of the referral system as possible. Comparing the cost-effectiveness ratios for the same health problem, the highest ratios were generally found in provincial hospitals followed by district level hospitals and outpatient care. According to calculations not presented in Table 1, cost-effectiveness ratios at health centres were also lower than hospital outpatient departments. In other words, this suggested that there could be substantial gains from utilising the public health care sector facilities in the hierarchical manner that was intended (e.g. only treating difficult health problems at high level facilities and mild cases at the lowest levels). Fourthly, there appeared to be a potential for designing very cost-effective preventive health interventions at the expense of curative interventions. The estimated costs per DALY for five preventive health programmes included in this study were all relatively low.

The sensitivity analysis presented in Table 4 suggested that increasing the discount rate to 6%, utilising the actual Zimbabwean life expectancies, applying equal age weighting or assuming a better capacity utilisation of health facilities had relatively minor effects on the cost-effectiveness ratios. As compared to the baseline estimates of Table 1, these assumptions resulted in a difference in costs per DALY of less than 20% for the majority of the 65 interventions included in the study. In addition, the majority of interventions had shifted their rank by three places or less in the rank order of interventions by cost-effectiveness ratio. Contrary to these observations, utilising a higher discount rate of 10% had more profound effects on the figures. Cost-effectiveness ratios of most interventions rose by 30% or more and the rank changed by four steps and above for 24 interventions. Reducing the estimates of effectiveness in individual health interventions, the rank of health interventions was more strongly affected at sufficiently large decreases in intervention effectiveness. For instance, if intervention effectiveness was 50% of the original estimates, more than half the interventions decreased their ranks by more than five places. In particular, the ranks of hospital treatment of bacterial meningitis, malaria, pneumonia, tuberculosis and surgical interventions as well as health centre treatment of conjunctivitis, schistosomiasis haematobium and gastroenteritis were sensitive to changes in intervention effectiveness.

**Table 4:** Influence of selected assumptions and parameters on the level of cost-effectiveness ratios and the rank order of interventions by cost-effectiveness ratios as compared to baseline estimates

Assumption or parameter investigated	Difference in costs per DALY as compared to baseline				Difference in rank order as compared to baseline			
	--------- change in percent ---------				------ places up or down ------			
		
	0–10	10–20	20–30	≥ 30	0–1	2–3	4–5	≥ 6
	---- Number of interventions ----				---- Number of interventions ----			
		
Discount rate 6%	55	0	4	6	47	13	3	2
Discount rate 10%	8	3	2	52	25	16	15	9
Zimbabwean life expectancies	20	38	3	4	57	7	1	0
Equal value on each life year	11	34	14	6	45	18	1	1
Capacity utilisation 80%	43	11	10	1	41	21	3	0
Capacity utilisation 95%	19	20	14	12	32	28	4	1
Effectiveness 90% of baseline	2	63	0	0	41	21	3	0
Effectiveness 70% of baseline	0	0	2	63	8	22	19	16
Effectiveness 50% of baseline	0	0	0	65	5	9	10	41

Note: The median costs per DALY utilising an alternative assumption or parameter were compared to the median costs of Table 1. Likewise, the rank order of interventions by median cost-effectiveness ratios utilising an alternative assumption or parameter were compared to the rank order of Table 1.

## Discussion

### Limitations of the approach applied in Zimbabwe

The above findings must be seen in the light of the limitations of the study. As a starting point, the cost function assumed for this study was simple and restrictive. The assumptions of constant returns to scale and perfect divisibility ensured that the average costs per unit of health of any given health intervention were the same irrespective of the level of production. These assumptions facilitated the identification of which interventions should go into an essential health package as well as the exact level of health services production necessary to eliminate the disease burden of a health problem. While there may be several reasons for deeming these assumptions unrealistic, they are frequently applied both for curative and preventive interventions [56-58]. Relieving the assumptions of constant returns to scale and perfect divisibility to develop cost functions with non-constant unit costs and limited possible production levels would require more complicated optimising techniques such as linear, non-linear or integer programming [59,60].

Much of the information necessary for costing of health interventions was available in principle in the sense that the majority of health facilities and vertical programmes visited kept a good record of most of the resources used although only at an aggregate level (i.e. total resources for a whole hospital and not broken down by diagnosis). However, the costing exercise was nevertheless in some aspects not as detailed and extensive as could have been desired mainly as a result of limited research resources. First, a relatively limited number of health centres, hospitals and preventive activities in districts were included as study sites leading to a risk of not capturing a representative pattern of unit costs for the country as a whole. Second, the costing methodology also involved a number of simplifying assumptions in the data collection at study sites including, for instance, that the average personnel and other costs per inpatient day were the same irrespective of the diagnosis of a patient and that the treatment of most conditions followed the treatment guidelines [23] rather than the pattern found through the micro-costing data collection. Third, the number of health interventions included was relatively low due in particular to problems of identifying sufficient information to estimate effectiveness of interventions.

Perhaps the most serious limitation was the scarcity of evidence on effectiveness of health interventions and of health systems research in Zimbabwe. The observations made by several authors [14,61,62] that we desperately lack data on these aspects are therefore also very relevant for the Zimbabwean situation. Research on factors influencing how health interventions work in the real world was extremely limited. One example of an extremely useful piece of research for the purposes of the present study was the finding of Vundule and Mharakurwa [63] that as many as 21 percent of villagers might refuse access of spraying teams to some rooms during residual house spraying. For the present study, the necessary information required to estimate effectiveness was for the majority of health interventions based on best guesses by clinical experts.

### Operational lessons learned

The present exercise replicated the methods underlying the 1993 World Development Report [16] and utilised also in more recent analyses [6]. The Zimbabwean research proved very data intensive and most of the needed information to determine the burden of disease, costs and effects of health interventions was not immediately available which required very extensive data collection in the field. Results presented here as well as the burden of disease estimates published previously [18] represented what was feasible within the time and resources allocated. The net time of work for this research was approximately one and a half year with one researcher, a study administrator and eight research assistants working full time and a core team of ten researchers and civil servants from selected ministries working on a part-time basis (3–6 hours per individual per week on average). Despite this substantial resource input, it was deemed necessary to adopt a number of simplifying assumptions and less extensive data collection activities some of which have been discussed above.

Substantial efforts were invested in searching for published and unpublished studies on the effectiveness of health interventions as well as health systems research on how health interventions operated in reality in Zimbabwe. The lack of knowledge was a main obstacle to this study and came to some extent as a surprise to research team. If this situation had been anticipated, the data collection would have focused more on collecting additional information on the functioning of the health system which could subsequently have been utilised to adjust the findings from efficacy studies measuring impact under ideal conditions to arrive at estimates of effectiveness for the Zimbabwean situation. Efficacy studies from similar settings in Africa are more plentiful [i.e. [64,65]]. One example of a possible useful piece of health systems research would be a review of the health centres of this study to investigate the compliance of tuberculosis patients on directly observed treatment short course (DOTS).

The characteristics described above had as a consequence that it was possible to include only a limited number of interventions for the cost-effectiveness analysis. In other words, cost-effectiveness assessments were not performed for a whole range of relevant interventions. For instance, interventions directed at health problems with a significant burden of disease such as depression and anxiety disorders and road traffic accidents were not included. Preventive interventions were incorporated only in a limited number so that many common HIV preventive activities were left out of the analysis including condom promotion, voluntary testing and counselling, peer-based programmes to educate high risk groups and prevention of mother-to-child transmission but also preventive activities against non-communicable diseases and injuries. As a result of these shortcomings, it is not possible to conclude that we have now identified the best or the most efficient essential package of care which must be focused on for many years. There may be other more efficient packages since it is possible only able to make conclusions with regards to reallocations among the interventions investigated in this study. The present endeavour may more appropriately be seen as just one possible direction towards improving allocative efficiency in the health care sector.

Nevertheless, the present exercise was still extremely useful. It captured costs per DALY of a range of health interventions representing the situation in the health care sector at the time of study. Before this study, there was only very limited and scattered information on the costs and even less on the effects of health interventions [55,66-68]. This study also demonstrated what was possible in a setting like Zimbabwe given a certain level of research resources but also that most of the limitations mentioned could in principle be overcome if more research resources were available. For instance, the cost data collection could have been extended and more health systems research could have been planned to inform the effectiveness component which could have enlarged the range of health interventions for the cost-effectiveness analysis. Finally, this study highlighted the most important gaps in the knowledge for priority setting i.e. the shortage of hard evidence on effectiveness of health services.

### Utilisation of results

According to the health information system, acute respiratory infections, malaria and skin diseases are the most common health problems treated at outpatient department level whereas pulmonary tuberculosis (as much as 22% of all inpatient days in the country), malaria and pneumonia are the most frequent diagnoses in individuals admitted as inpatients at hospitals in 1998 [19]. Large proportions of pulmonary tuberculosis and pneumonia cases are probably caused by underlying HIV [18]. Most of these interventions have been deemed relatively cost-effective in this study and are components of the packages suggested (Tables 2 and 3). However, the present study suggests that HIV prevention is more cost-effective than treatment (including TB treatment) which has been confirmed by other studies [69]. Also malaria prevention in high incidence areas appeared to have lower costs per DALY compared to treatment. The central level of Ministry of Health controls the overall allocation of funds between hospitals, where the majority of interventions are curative, and preventive programmes. It is therefore possible to change priorities at the macro level by shifting the balance in favour of the preventive component and furthermore to focus on HIV and malaria prevention. Apart from deciding the funds available for individual hospitals, the central level of Ministry of Health has less influence on the priority setting at health centre and hospital level where the interventions offered to a large extent are determined by self-referral of patients. Rationing decisions for treating different patient groups will be done by health practitioners and may involve a variety of considerations and values (see i.e. [70]) resulting in a focus which may differ from priority setting based on pure cost-effectiveness criteria. The present study pointed to a direction of focus among curative health interventions which may be difficult to enforce among health practitioners. Increased legitimacy and support among clinicians and other health sector personnel as well as patients may be secured if they are involved in the priority setting procedure through a consultative process where these groups are allowed to incorporate their own values. For instance, several authors have suggested that other criteria such as equity, severity of disease, age of patient groups and capacity to benefit may affect the rank order of health priorities in the opinion of health personnel [71-73].

## Conclusion

The previous pages showed that it was feasible to conduct cost-effectiveness analyses for a large number of health interventions in a developing country like Zimbabwe using a consistent methodology similar to the analysis performed at a general, non-country specific level by the World Bank [16]. The analyses performed in Zimbabwe suggested that cost-effective health interventions were available for some of the major health problems including HIV, pneumonia, tuberculosis and malaria. In addition, the analysis suggested that there was substantial potential for improving the efficiency with which resources are utilised in the public health care sector.

Limitations to the approach applied in Zimbabwe were identified including short cuts in the costing methodology and scarcity of evidence on effectiveness of health interventions. As a result, important health interventions were not incorporated in the cost-effectiveness analysis. However, most of the obstacles identified in this study could in principle be overcome by adding more research resources. For instance, adding a large component of health systems research on the actual functioning of the health system would improve the effectiveness estimates and enable the inclusion of more health interventions in the cost-effectiveness analysis. A larger number of health interventions assessed by cost-effectiveness analysis would in addition make the subsequent identification of an essential package of health interventions more credible.

## Competing interests

The authors declare that they have no competing interests.

## Authors' contributions

KSH contributed substantially to the conception and design of the study, data collection in the field, analysis of the data and drafted the manuscript. GC contributed substantially to the conception and design of the study, data collection in the field, analysis of the data and critically reviewed the manuscript. All authors read and approved the final manuscript.

## Annex 1

See Table 5.

**Table 5:** Annex 1: Total burden of disease as measured by Disability-Adjusted Life Years (DALYs) and distribution by the top 25 underlying causes, Zimbabwe, 1997

Total DALYs	4,948,172
Cause	%
HIV	48.6
Depression and anxiety disorders	5.6
Diarrhoeal diseases	3.5
Low birth weight	3.4
Lower respiratory tract infections	3.3
Birth asphyxia and birth trauma	2.7
Protein-energy malnutrition	2.3
Malaria	1.8
Tuberculosis	1.6
Road traffic accidents	1.1
STIs excluding HIV	1.0
Iron-deficiency anaemia	1.0
Bacterial meningitis	0.9
Maternal sepsis	0.9
Sense organ diseases	0.9
Self-inflicted injuries	0.8
Cerebrovascular disease	0.7
Endocrine disorders	0.6
Alcohol dependence	0.6
Rheumatic heart disease	0.6
Obstructed labour	0.5
Hypertensive heart disease	0.5
Diabetes mellitus	0.5
Asthma	0.5
Inflammatory heart disease	0.5
	
All other conditions	15.8

Source: [18].
